# Nurses’ Fall Prevention Interventions in Nursing Home Patients With Acute Versus Chronic Underlying Conditions

**DOI:** 10.1093/geroni/igaa057.763

**Published:** 2020-12-16

**Authors:** Deanna Gray-Miceli, Alison Kris

**Affiliations:** 1 Thomas Jefferson University, Philadelphia, Pennsylvania, United States; 2 Nursing, Fairfield, Connecticut, United States

## Abstract

Nursing Home (NH) nurses care for over 1.6 million older residents each year. Among this vulnerable population, an estimated 50 percent of older residents fall each year. Although licensed nurses caring for NH residents who fall intervene to prevent fall recurrence, we know little about nurse’s perceptions of the most effective interventions for various types of falls they manage. The purpose of this qualitative study is to describe and compare licensed nurse’s perceptions of fall prevention interventions believed to be due to acute underlying causes of a fall versus those believed to be due to chronic underlying conditions. This study is a secondary analysis of existing qualitative data from a multi-site parent study conducted in three NH sites in the northeastern U.S. designed to test nurse’s knowledge of falls prevention and interventions. Forty seven registered or licensed practical nurses, English speaking who were full or part time employees were recruited to participate. Most were female (n=46; 98.7%) with a median age of 49.5 years and ten years’ experience. Using Colazzi’s (1978) method, 47 responses of nurse’s were read from typed transcripts and analyzed independently by 2 judges. Significant statement were extracted to derive meanings and form themes. For falls related to acute causes, nurses most often stated they would collaborate with the physician, propose a blood pressure intervention and promote safety. For falls due to chronic causes, nurses promoted ambulation safety, pain interventions and collaborated with specialists. Since nurses intervened differently, identifying fall type is critical in selecting appropriate interventions.

